# The Arsenization Idea

**Published:** 1905-09

**Authors:** 


### The Arsenization Idea.

Some difficulty has arisen from the notoriety attending the visit to New Orleans of Dr. Reginald B. Leach, a homeopathic physician of St. Paul, Minn., who holds that arsenization is a preventive of yellow fever infection. He has talked freely to the newspapers and has addressed public meetings of sympathizers. He announced that he came to New Orleans to give his theory a practical demonstration, by allowing himself to be bitten by an infected stegomyia. He refused to go on with the experiment unless it was conducted by the New Orleans Medical Society, which refused his request, not caring to countenance an experiment that might result in death, and not having any active members who could well be spared from the active fight against the disease. Many physicians fear an epidemic of arsenical poisoning, the popular love for a "specific" having led hundreds, after reading Dr. Leach's newspaper interviews and articles, to eat arsenic. Belief in this extraordinary theory has led many citizens of New Orleans to ignore the orders of Dr. White and his assistants. The eaters of arsenic pills have neglected to clean up their premises and screen their cisterns. Orders were issued on August 18th for the instant arrest of all who have failed to obey the mosquito ordinance. The disciples of Dr. Leach held an indignation meetnig at which the medical profession and the press were jointly denounced.—*Journal A. M. A.*

